# The Complementary Roles of Neurological and Musculoskeletal Physical Therapy and Regenerative Medicine: A Comprehensive Review

**DOI:** 10.3390/medicina60071062

**Published:** 2024-06-27

**Authors:** Maryam Mureed, Arooj Fatima, Tayyaba Sattar, Syeda Aiman Batool, Ambreen Zahid, Haleema Usman Khan, Arooj Fatima, Hamna Shahid, Saba Nasir, Mehsn Yizdin, Elih Tehmahb, Hamid Tebyaniyan

**Affiliations:** 1 The University of Lahore, Lahore 54570, Pakistan; maryamhaider002@gmail.com (M.M.); haleema321842@gmail.com (H.U.K.); hamnashahid997@gmail.com (H.S.); 2 University Institute of Physical Therapy, University of Lahore, Lahore 54570, Pakistan; arooj.fatima1@uipt.uol.edu.pk (A.F.); tayyaba.sattar@uipt.uol.edu.pk (T.S.); abatool902@gmail.com (S.A.B.); 3 Institute of Physical Therapy, University of Lahore, Lahore 54570, Pakistan; ambreenusman95@gmail.com; 4 Islam Medical College, Sialkot, Pakistan; aroojhaider12@yahoo.com; 5 Forman Christian College University, Lahore 54600, Pakistan; nasirsaba2442@gmail.com; 6 Department of Science and Research, Islimic Azade University, Tehran 14878-92855, Iran

**Keywords:** regenerative medicine, physical therapy, tissue repair, rehabilitation

## Abstract

Regenerative medicine, encompassing various therapeutic approaches aimed at tissue repair and regeneration, has emerged as a promising field in the realm of physical therapy. Aim: This comprehensive review seeks to explore the evolving role of regenerative medicine within the domain of physical therapy, highlighting its potential applications, challenges, and current trends. Researchers selected publications of pertinent studies from 2015 to 2024 and performed an exhaustive review of electronic databases such as PubMed, Embase, and Google Scholar using the targeted keywords “regenerative medicine”, “rehabilitation”, “tissue repair”, and “physical therapy” to screen applicable studies according to preset parameters for eligibility, then compiled key insights from the extracted data. Several regenerative medicine methods that are applied in physical therapy, in particular, stem cell therapy, platelet-rich plasma (PRP), tissue engineering, and growth factor treatments, were analyzed in this research study. The corresponding efficacy of these methods in the recovery process were also elaborated, including a discussion on facilitating tissue repair, alleviating pain, and improving functional restoration. Additionally, this review reports the challenges concerning regenerative therapies, among them the standardization of protocols, safety concerns, and ethical issues. Regenerative medicine bears considerable potential as an adjunctive therapy in physiotherapy, providing new pathways for improving tissue repair and functional results. Although significant strides have been made in interpreting the potential of regenerative techniques, further research is warranted to enhance protocols, establish safety profiles, and increase access and availability. Merging regenerative medicine into the structure of physical therapy indicates a transformative alteration in clinical practice, with the benefit of increasing patient care and improving long-term results.

## 1. Introduction

Regenerative medicine and physical rehabilitation sciences are combined in regenerative rehabilitation. While physical therapy is fundamental for restoring or revitalizing function to injured tissue, its targeted influence on cellular-level regeneration is relatively unknown. At the same time, within regenerative techniques, the mechanical conditions affecting cells and scaffolds subjected to orthopedic repair are typically perceived as an obstacle to be persevered through or navigated instead of being seen as a potential gain to be optimized [1,2,3,4]. Meanwhile, tissue engineering tends to concentrate on cellular mechanobiology to boost the maturation and spatial arrangement of engineered assemblies, such as the arrangement of muscle fibers and the layered structure of chondrocytes. Regenerative rehabilitation can be viewed as a method for applying translational mechanobiology, where mechanical stimuli influence the regulation of cell differentiation, and function may be leveraged by rehabilitation routines to facilitate restoration and regeneration [1,5,6,7]. In regenerative medicine, a cross-disciplinary domain, replacements for cells, tissue, or organs are adopted for the purpose of strengthening and amplifying the body’s healing capabilities. Considering the main purpose of these therapies is to reestablish typical function in impaired or infected tissues, the trajectory of regenerative medicine is inextricably linked to the future of rehabilitation [8,9,10,11]. Rehabilitation specialists ought to keep abreast of the latest innovations in regenerative medicine and work in tandem with basic researchers to help in crafting the evolution of practical clinical protocols. The objective of the present paper is to discuss the modern outlook on biological approaches for managing musculoskeletal disorders, along with focusing attention on the incorporation of regenerative medicine with physical therapeutics [8,12,13,14]. The opportunity for absolute healing of damaged or degenerated musculoskeletal tissues is demonstrated by regenerative medicine, contributing hope for patients with conditions previously proven to have limited recovery prospects. Various issues with the musculoskeletal system are aided by regenerative medicine, together with injury-related issues that pivot on repair for healing, for instance, muscle strains, ligament sprains, tendon ruptures, and skin wounds. Furthermore, injury-related conditions that recuperate slowly, including osteochondral defects and nonunion bone fractures, might also benefit from the support of regenerative medicine, as might injury-related conditions with minimal healing potential, for example, substantial muscle atrophy and bone section gaps. Medical conditions related to disorders like sarcopenia, osteoporosis, and osteoarthritis may also be dealt with using regenerative medicine [15,16,17,18,19,20,21]. Presently, these conditions are treated using some regenerative therapies, which include the administration of stem cells, progenitor cells, or biologically active molecules together with engraftment of bioengineered scaffolds or in vitro-grown tissues. This article delivers insights into the impact of mechanotherapies on several tissues, more specifically, bone maturation and repair [15,22,23]. The process of repair and recovery of musculoskeletal tissues caused by implementing regenerative medicine therapies relies on the native tissue accepting the therapy and successful mucosal tissue development by enhanced mechanical properties. Mechanotherapies, in combination with regenerative medicine treatment, offer substantial promise for synergistic results and cumulative outcomes once applied by physical therapists [15,24,25]. A physical therapist intends to induce a tissue response at a cellular and molecular level by forming a force. This force is created by mechanical stimuli, which can be either extrinsic or intrinsic. The issue that arises is related to the capacity of patients that need regenerative therapy, as their load-bearing ability is bound, and hence, such therapy may be damaging to the health of individuals. Using microscopic cell study and having a thorough understanding of the concepts of the tissue forces around it may allow the introduction of innovative and groundbreaking methods of mechanical forces at optimal levels [15,26,27]. The teamwork of physical therapists and scientists may allow the creation of pioneering medical breakthroughs for treating musculoskeletal disorders by evolving traditional practices, such as replacing prosthetics with regrowing an amputated limb, most likely in a manner akin to salamander limb regrowth after amputation. Similarly, new strides in regenerative medicine imply attention toward physical therapeutics as a potential option for the treatment and management of musculoskeletal diseases and injuries using accomplished biological therapies [8,12,13,17]. The hypothesis of regenerative rehabilitation arose to address this difficulty. In summary, regenerative rehabilitation is defined as “the protocols and principle implementation of rehabilitation in conjunction together with regenerative medicine therapeutics in order to maximize functional restoration by means of tissue regeneration, restructuring, or cellular repair”. A combination of such regenerative rehabilitation and regenerative therapy techniques, which commonly are not combined, assists in the promotion of tissue rejuvenation and functional restoration [4,14,24,28]. Regenerative medicine techniques grouped with rehabilitation facilitate optimal stimulation and protection of transplanted (donor) cells in addition to creating the optimal environment and initiating and maintaining recipient cells, which are the host cells, thus encouraging the expedition of the process of tissue regeneration. Regenerative rehabilitation improves standard rehabilitation practices by focusing on and improving the patient’s ability to resume daily tasks and reintegrate into society. Advancements in rehabilitation solutions are pursued as a result of the latest breakthroughs in robotics and comparable fields [2,13,18,25,27,28]. This review article reviews the impact of regenerative medicine in the context of physical therapy.

## 2. Study Design

This comprehensive review seeks to explore the evolving role of regenerative medicine within the domain of physical therapy. A checklist-based strategy was used for the search, screening, and data extraction. Six reviewers (Maryam Mureed, Arooj Fatima, Tayyaba Sattar, Syeda Aiman Batool, Ambreen Zahid, Hamid Tebyaniyan) independently searched Web of Science, Scopus, Pubmed, and Google Scholar for relevant articles. The search terms “Regenerative medicine”, “Physical therapy”, “Tissue repair”, and “Rehabilitation” were used to find all relevant publications up to April 2024. The first step was to search and retrieve studies. Duplicates were discarded; afterward, the papers’ titles and abstracts were vetted.

Study inclusion criteria were then applied to those remaining articles to finalize the screening process. The reliability between the evaluators regarding the literature screening process was calculated using Cohen’s kappa coefficient. Based on the frequency of precise agreement between reviewers, the kappa value (k) was determined. In vivo, review, and original studies describing the role of regenerative medicine in physical therapy were included. In vitro studies, posters, non-English language studies, abstracts, and studies with insufficient data were excluded. After that, 11 reviewers (Maryam Mureed, Arooj Fatima, Tayyaba Sattar, Syeda Aiman Batool, Ambreen Zahid, Haleema Usman Khan, Arooj Fatima, Hamna Shahid, Saba Nasir, Mehsn Yizdin, Elih Tehmahb) extracted the required data. An author (Hamid Tebyaniyan) was consulted in cases of disagreement among reviewers.

The initial search resulted in 280 articles. Upon removing duplicates, 165 remaining studies were reviewed based on title and abstract. A total of 93 records were excluded after analyzing the full text of articles. A total of 72 studies were included to structure this review. Based on Cohen’s kappa coefficient, the evaluators were in almost complete agreement.

## 3. The Convergence of Regenerative Medicine and Physical Therapy

The principles, techniques, and strategies from regenerative medicine and rehabilitation are combined in regenerative rehabilitation to elevate their configuration and role and improve patient health challenges [29,30,31,32]. The method of regenerative science medicine for replacing either injured tissue or, alternatively, a whole organ consists of the use of cells and biologics, the utilization of a combination of these strategies and approaches, or both biomaterials and scaffolds. Although regenerative medicine’s therapeutic and clinical potential is significant, regenerative rehabilitation offers the chance to impact regenerative medicine favorably by integrating rehabilitation sciences principles, as regenerative medicine alone cannot guarantee the prosperous application of strengthening the functioning of the targeted organ of patients [3,6,29,33]. However, with deep comprehension and deeper insights into concepts of regenerative medicine, rehabilitation researchers can more effectively modify and adjust rehabilitation attempts to optimize the enhancement of regenerative methods. Several different regenerative rehabilitative techniques can comprise exercise-stimulated plasticity, physical training prescription, electrostimulation, and the incorporation of nutritional boosters. The preclinical studies step is extremely important to carry out, as the translation of regenerative medicine techniques to human applications may be challenging and, if skipped, may result in severe outcomes in rehabilitation studies, such as in issues of mobility, articulation range, stimuli response, and ache. Collaboration and teamwork among scientists, researchers, and clinicians across the spectrum of disciplines are proposed by the authors to optimize the reestablishment of function and living standards for individuals with impairment and trauma [4,13,25,29,33,34]. The precise implementation of mechanical stimuli forms the foundation of physical rehabilitation and encourages the enhancement of the prospects of intrinsic tissue repair. Mechanobiology, being an expanding scientific field, focuses on comprehending the influence of physical forces to elicit responses in cells and tissues, as well as the effect of these forces on tissue maturation, homeostasis, and disease mechanisms. At the core of mechanobiology lies mechanotransduction, which is how mechanical signals are registered, replayed, and transformed into biological reactions [35]. Biological adaptations as a reaction to both moving and stationary mechanical forces are strongly supported by evidence. Modifications in cellular dynamics, extracellular matrix (ECM) structure and components, and mechanotransduction processes might be implicated in the disease progression of several inherited and acquired disabling health issues according to progress in mechanobiology. The natural healing potential of tissues can be harnessed by the application of mechanical stimuli, which act as a powerful catalyst [23,34,35]. This concept has laid the groundwork for using rehabilitation protocols to treat diseased or injured tissues. Corresponding mechanical and biological stimuli contribute to nervous system stimulation, which then promotes reorganization and potential repair. Many therapies aim to enhance neuroplasticity, which is commonly achieved by combining physical movement with nervous system activity. The recovery process is supported by modern physical therapy approaches through the use of supported motion and activation of the brain, muscles, spinal cord, or nerves, making use of this activity-dependent plasticity. In the future, pairing these active, timing-dependent methods using stem cell or tissue engineering innovations will be necessary to direct tissue graft integration or foster the revival and functional structure of intrinsic stem cells [14,27,35].

### 3.1. Neurological Regeneration

Electrical and chemical signals are thought to predominantly influence the plasticity and remodeling of the central nervous system (CNS). Subsequent to CNS injury, the development of fibrosis might change the tissue’s biophysical factors, potentially instigating downstream cellular responses that majorly impact restoration and plasticity. The steady mechanical and electrical features of the cell’s immediate environment strongly affect the regenerative capabilities of mesenchymal stem cells. Neurons from the spinal cord and hippocampus are cultured on a gel-based substrate format that is softer, and they have triple the amount of branches as those developed on stiffer gels [5,18,24,35,36]. These studies, collectively, indicate that using complementary approaches to enhance the biophysical microenvironment may be essential in completely achieving the efficacy of rehabilitation strategies postspinal injury, -stroke, or -brain trauma. Various techniques exist for triggering both the brain and spinal cord using electrical or magnetic stimulation after injury. Electrical stimulation, also known as epidural stimulation, is a method that involves current directed to the upper surface of the spinal cord. Early human trials are feasible because of the off-protocol method application of inducers originally intended to relieve severe pain [7,12,35]. Magnetic fields offer noninvasive spinal stimulation methods that have been shown to improve muscle stiffness after spinal cord injury for up to 24 h. Magnetostimulation situated in the lumbar spinal cord could be induced through the motion of the upper arm, establishing an action-based framework at which point stride actions align with arm oscillation in research participants with uninjured, fully functional spinal cords. A damaged central nervous system requires proper neural activity prior to stem cells to enhance functionality, as neural activity is crucial in averting cell death after injury, augmenting better blood delivery and neuronal vitality, and elevating brain-derived neurotrophic factor (BDNF) concentrations, which happens to be linked to with plasticity and renewal [5,6,35]. In spinal muscular atrophy models, standout activity corresponds with decreased axon growth. Based on this evidence, short-duration electrical stimulation sessions of the peripheral nerve were combined alongside motor neuron cell implants in a leading study about stem cell transplant, leading to remarkable cell persistence and function of muscle reinnervation. This pivotal study indicates that the integration of regenerative cell therapies with artificial stimulation may be crucial to attaining the plasticity and the functional restoration that were desired postinjury to or -deterioration of the neuromuscular system [27,35]. Stem cell transplantation can hold a long-lasting, additional, enduring supply of neuroplasticity. In this context, the term neuroplasticity denotes the structural or function modification of a neural system triggered by a cue [37]. Thus, subsequent to the wound, stem cells can be bolstered structurally or functionally towards a new neural substrate, presenting a pathway to apply rehabilitation methods to enhance the effectiveness of modern existing transplantation methods. The use of combined therapies is advocated along with sensory input stimulation or motor activity stimulation on the nervous system through injury. The foundation for maximizing the opportunity of innate stem cell-driven recovery through rehabilitation is to outwardly apply sensory and motor stimuli to trigger the reaction of stem cells [18,33,36,37]. Investigations of the dynamics of sensory feedback and motor execution on hippocampal neurogenesis discovered that sheltering animals in a stimulated and enriched environment contributed to the longevity of newly born hippocampal neurons in due course. Furthermore, self-directed wheel-running exercise brought about a substantial rise in new hippocampal neuron formation and bolstered the animals’ chances of survival. Older animals on the running wheel with an average daily distance of 3.9 km (SD = 0.1) restored the inherent diminishment in hippocampal neurogenesis by 50%. In light of this, endogenous stem cells may be triggered for a suitable response if the stimuli are timed and dosed optimally. Likewise, for the more substantial and consistent functionally active result, the same stimuli could be used to support transplantation by improving donor cell vitality, future, and synthesis. The outcomes of stem cell reaction pathways to conventional exercise and optimized living conditions are being acknowledged. Teng’s team suggested that exercise impacts the morphology of dendritic cells, boosting vascular development and modifying synaptic and receptor protein production anatomically and physiologically. This adaptability can have a direct impact on neural stem cell reservoirs. On the flip side, there may be negative outcomes of activity or physiological practices in the affected area. The emphasis of physical rehabilitation is usually neuromuscular system initiation, potentially at the cellular level, with the intention of improved outcomes and enhanced behavioral alterations. In view of this, stem cell technologies effectively collaborate with rehabilitation. Authors of the latest review emphasized the considerable potential and promising outcomes of using solitary strategies of CNS regenerative medicine and merging neurorehabilitation approaches and how these should be more generally adopted for superior results. Drawbacks concerning the present topic were brought to attention, including the organized input given through means over a considerable period that may restrict cell replacement therapies by acclimating transplanted cells and comprised sites for survival [37].

### 3.2. Musculoskeletal Regeneration

Musculoskeletal regenerative rehabilitation is noted as the incorporation of concepts and methods from rehabilitation and regenerative medicine by way of the final objective of encouraging functional recovery through musculoskeletal tissue regrowth and repair. Physical therapists are not restricted to the restoration of tissue function after its regeneration but are free to contribute significantly in aiding tissue regeneration while the tissue heals [2,3,4,15]. This review discusses the important role therapists play in the development of novel regenerative therapies. This can be achieved by promoting the collaboration of various disciplines in relation to regenerative medicine, fostering a team-based approach to maximize functional effects. Success in regenerative medicine relies on the ability of therapies to revive or regenerate musculoskeletal tissues. These therapies must be received and incorporated into native tissues to develop musculoskeletal tissues with refined mechanical traits. A consolidated group, above all of therapists, that has a repertoire to exhibit substantial opportunities comprises physical therapists who may show potential outcomes on affiliating their skillset with regenerative medicine treatment by performing mechanotherapy [5,6,15,34].

To date, the prime achievement in the field of regenerative rehabilitation research has been observed in musculoskeletal applications, specifically in traumatic skeletal muscle injury care. In spite of the fact that notable regenerative potential is seen, skeletal muscles are restricted to severe, extensive injuries or conditions that damage the foundational architecture and impede the regeneration process, influencing tissue-scarring formation [7,12,13,35,38,39,40]. As a consequence, the functional capacity of such individuals’ damaged tissue is significantly reduced, so cellular therapies are considered to enhance the tissue’s regenerative ability. Frequently, these treatments face constraints due to significant cell loss post-transplantation and limited transplant success rate, resulting in suboptimal functional outcomes. Researchers use a practical resolution to mitigate this problem by combining stem cell transplantation with muscle strengthening exercise to improve cell engraftment of the donor in cases of both muscle trauma (myopathy) and injury [14,16,35]. Positive tissue reconstruction in cases of volumetric muscle loss (VML) has been reported to be assisted by the implantation of cell-free biologic scaffold materials consisting of mammalian ECM. Although the precise mechanisms accountable for perceived functional improvements are yet to be completely clarified, assumptions about attracting stem/progenitor cells at implant sites by donor ECM are held to be true. Rehabilitation protocols subsequent to ECM implantation have been proposed as advantageous, and even essential, for delivering the mechanical signals crucial for precise tissue remodeling. Additional randomized trials are required to examine how optimized rehabilitation protocols could improve functional results after the use of tissue engineering devices for the treatment of VML [17,35]. Mechanical forces are applied in practically every physical therapy in musculoskeletal rehabilitation regardless of the type of forces used: extrinsic force generated by therapist intervention or force intrinsically generated by the patients through prescribed exercise therapy. Not all the various mechanotherapies adopted by physical therapists are covered in this article. Alternatively, readers are guided to the following reviews, which provide comprehensive information on different physical interventions such as articular mobilizations, muscle and tendonous stretching, strength exercises, vibration therapy machines, therapeutic ultrasound, and massage therapy [15,33]. Bone restructuring is externally stimulated by low-intensity vibration [41], as advocated by Rubin and colleagues, whose initial research reported a 34% rise in bone density of the proximal femur trabecula in adult sheep compared to control subjects when exposed to LIV stimuli with a force of lower than 0.3 g (where g = earth’s gravitational field) alongside a frequency of 30 Hz for 20 min daily throughout a year [42].

## 4. Regenerative Medicine

Methods for restoration, repairing, advancing, or regenerating damaged cells, organs, and tissues as a result of aging, illness, prolonged disorders, or congenital disorders are achieved through the integration of various disciplines in regenerative medicine. Novel therapies that rejuvenate structural and biological function post-tissue trauma and decelerate disease progression are the purpose of regenerative medicine. Novel treatment strategies, including tissue regeneration, cytobiology, and biomaterials, are brought forth by this field [18,36,43]. ECM or tissue support can play a vital role in directing the host’s recovery and healing process in special cases of pronounced tissue impairment by supplying structural and biochemical support under tissue engineering. This strategy causes a biological induction called a three-dimensional construct. A prime example of such a situation is a large gap as a result of peripheral nerve injuries that hinders the rejoining of residual nerve buds; hence, the separation impedes regeneration into the original operational configuration. This is where inserting a scaffold opens a passage for connection between nerve ends, which has been proven to significantly enhance the healing process in animal studies. A scaffold must have two major defining qualities: firstly, biological stimulation attributes required for the host’s healing process, and secondly, fast biodegradability in the body [8,22,23]. The core purpose of regenerative medicine is to reestablish lost or impaired tissues to a condition that is fully operational and visually indistinguishable in appearance from the tissues preinjury. This encompasses not only regenerating defined tissues, namely, nerve, muscle, skeletal tissue, skin, and blood vessels, but also ensuring flawless blending into the nearby surrounding tissue. Barring vascularized composite allotransplantation, the restructuring utilizing matching tissue by means of autogenous tissue regeneration continues to be an elusive objective, using an assortment of tissues at various developmental stages. Currently, the leading FDA-approved therapy is bone regeneration used in clinical settings; although a variety of skin regeneration technologies have been established, the ultimate outcomes are less than ideal. Clinical trials are in the pipeline for regenerating broad-diameter arteries [24,29].

### 4.1. Regenerative Biomaterials

Regenerative materials that support the process of tissue regeneration consist of bioactive ceramics; naturally derived polymers like chitosan, hyaluronic acid, and collagen; and artificial polymers, for instant polycaprolactone (PCL) and poly(lactic-co-glycolic acid) (PLGA). Such biomaterials exhibit proven promise for restoring bone and cartilage, together with regenerating nerves, and achieve this by generating a targeted setting that boosts regenerative proficiency and healing capacities concerning dual, transplanted, and host cells. The scaffold pore structure guides the chondrogenesis and endochondral bone maturation in connection with bone marrow-derived mesenchymal stem cells (BMSCs), coupled with stimulating angiogenesis. The differentiation of myoblasts into myotubes is achieved with the assistance of an extracellular matrix hydrogel arrangement [13,25,44,45,46,47,48]. Within the body, osteogenic mineralization and the boosting of the multiplication of cells are achieved by using patches with nanotopographic features based on PCL, which aligns with the raised nanometer-scale matrix, plus indented patterns at about 800 nm and nanometer-scale pores. The effectiveness and safety of various biomaterials have been assessed, including those that enclose PCL microfiber scaffolds, hydrogels presenting collagen peptides, and adipose mesenchymal stem cell-derived structures. In a rat model, the issue of osteoarthritis, which stemmed from joint cartilage and knee joint pad deterioration, was resolved within eight weeks by establishing a lubricating barrier on the cartilage; hyaluronan-based scaffolds were encased in nanofibrous polymers designed to mimic biomimetic brushes. Moreover, tissue regeneration effectiveness was commonly observed when ECM scaffolds utilizing aptamer HM69, a viscoelastic network made from PEGylated poly(glycerol sebacate), were paired with osteoinductive mesoporous bioactive glass (MBG), in addition to BMSC-laden multiphasic biological mimicking [13,26,49,50,51]. Increased improvement in the healing outcomes of shoulder pain, like rotator cuff injury, was observed when a scaffold with gradient architecture that consisted of anisotropic properties was used, as it resembles a multistate microstructure. The potential for boosting regeneration and controlling inflammation in affected tissues is held by these new biomaterials that eliminate pain symptoms related to these conditions. Consequently, they can fill the role of an effective alternative to existing pharmaceutical pain management therapies [13,27].

### 4.2. Stem Cell Therapy

Over the past decade, tissue regeneration for stem cell injections has garnered significantly more attention. This method captivates the interest of many because stem cells are able to differentiate into different types of specialized musculoskeletal tissues, such as muscles, bones, cartilage, tendons, and ligaments. The accomplishment of stem cell therapy post-stem-cell administration is determined by several factors, including the stem cells’ longevity and division, transport to the damaged area, and differentiation into the targeted tissue type. Although in-depth research is scarce on post-stem-cell-therapy rehabilitation protocols, physical exercise is indicated by a number of studies to directly affect the initiation, movement, and differentiation of multiple stem cell types [3,16]. The introduction of cells is typically into a tissue through cellular therapies to attain an aimed-for effect. The main objective is to restore the functions of tissue involved in tissue repair that are impaired due to injury, aging, or illness [4,8]. The host’s regenerative response can be triggered by transplanted cells as they can play the role of pharmacological agents by stimulating the secretion of cytokine or exertion of paracrine effects. Rather than just inducing the host’s cells using stimulating regenerative responses or signals, the donor cells may incorporate with the host tissue and embody the role of replacing or restoring the damaged tissue themselves. As a case in point, aged skeletal muscle is characterized to exhibit lesser healing potential due to considerable reduction in the production and release of vital cellular growth regulators like vascular endothelial growth factor (VEGF), along with a reduction in regeneration as the muscle stem cells decline. These combined deficits lead to greatly reduced healing potential in aged muscle. Cell-based strategies offer hope, as transplanting cells could revive the regenerative capacity of aged muscle in two possible ways, including boosting secretion within a local area of essential growth factors and replenishing the collection of regenerating cells [8,14]. The aftereffects of spinal cord injury (SCI) can be attended to through the cutting-edge approach of stem cell-based regenerative therapy, as several clinical and preclinical studies have reported prominent treatment successes during acute and subacute stages, making use of the wide range of cells, among which are neural stem cells (NSCs), neural progenitor cells (NPCs), mesenchymal stem cells (MSCs), and olfactory ensheathing cells (OECs). Different therapeutic processes, like neuronal cell replacement, remyelination, and transplanted cells offering trophic enhancement, have been uncovered by researchers, contributing to the protection of tissues along with improving neuronal plasticity [22,52]. Cell therapies are most beneficial during the acute-to-subacute phase, as the following phases offer only limited benefits for long-standing injury of the spinal cord, mainly because of its inhibitory microenvironment. The use of stem cell therapy is subject to disagreement for treating chronic SCI patients due to the considerably high probability of anticipated negative side effects, for example, high temperature, infectious disease, perceptual irregularities, and muscle fatigue with risk of tumor genesis pertaining to cells harvested from stem cells that come from embryos and instigated pluripotent stem cells. Some investigations are underway that combine numerous treatments to attain noteworthy functional restoration in individuals dealing with chronic SCI. Although rehabilitation is frequently brought together with stem cell therapies within human subjects, the concurrent utilization of both in preclinical research is only in the initial stages. Regenerative rehabilitation, which refers to the coordination of rehabilitation with stem cell therapy, has received considerable acclaim as a secure, feasible, and results-driven method [3,4,52]. 

In the latter part of the 20th century, research shed light on the distinctive features of stem cells, revealing their ability to self-renew and differentiate in reaction to the external stimuli they are set in. Through bone marrow transplantation studies, researchers started to grasp how stem cells evolve, differentiate, and specialize into distinct cell types. Based on the developmental ability of a stem cell into a new line, these cells are categorized as totipotent, pluripotent, multipotent, and unipotent [4,14,53]. The process of differentiation in stem cells as they develop and mature results in them losing their self-renewing quality. However, initiatives are in progress to preserve the stem cells in their original state by creating an environment that aids an undifferentiated phase of cells. Pluripotent embryonic stem cells used for stem cell therapy have drawn criticism as embryo destruction is a part of the process; particularly, blastocyst-stage embryos are used for cell derivation, which has prompted adult stem cell exploration as there are multipotent stem cells situated in adult tissues and organs. An influential role of adult stem cells includes supporting physiological processes by swapping out dying cells or cells experiencing functional impairment in tissues or organs [22,53]. To avoid controversy, adult stem cells hold an edge compared to other stem cell therapies since cells are sourced from diseased patients or patients with cell/tissue loss; hence, their use only requires the patient’s consent. This is a major reason that extended research on adult stem cells is garnering more interest. Among the adult stem cells, notably, hematopoietic stem cells (HSCs), and MSCs are the most commonly used, primarily due to the fact that cell collection is from patients experiencing medical conditions. The key qualities and features needed for the implementation of adult MSCs in regenerative medicine and tissue engineering are elaborated in the upcoming sections [13,14,53].

**Figure 1:**
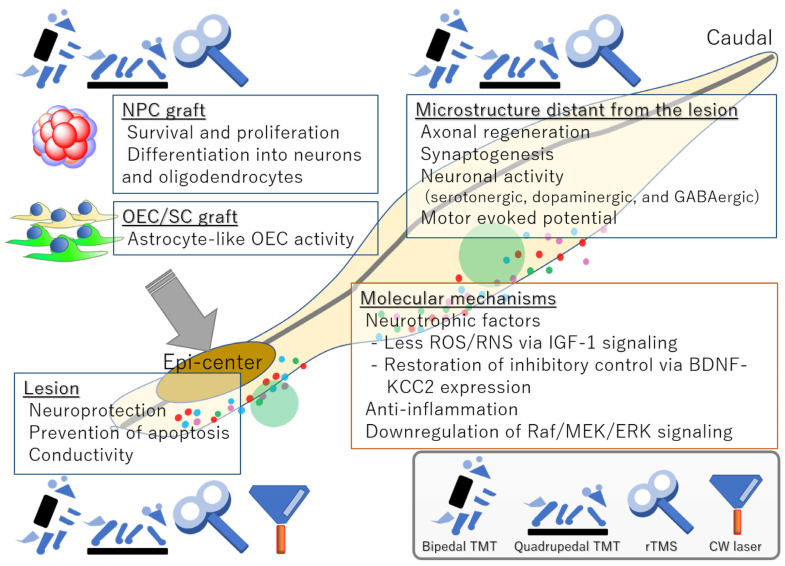
An illustrative overview of the impacts brought about by rehabilitation and regenerative treatment combination. BDNF: brain-derived neurotrophic factor; C.W.: continuous wave; IGF-1: insulin-like growth factor 1; KCC2: kalium-chloride cotransporter 2; OEC/SC: olfactory ensheathing cell/Schwann cell; ROS/RNS: reactive oxygen species/reactive nitrogen species; rTMS: repetitive transcranial magnetic stimulation; TMT: treadmill training [52].

Significant differences between clinical and preclinical studies are evident in the progress of regenerative rehabilitation. In clinical settings, healthcare providers often offer rehabilitation to patients with SCI, and patients regularly take part in continuously building capacity to maintain the extent of their joint movement, muscle strength, mobility, and day-to-day activities [36,52]. Additionally, numerous clinical environments correspond to the successful administration of rehabilitation, hence making it a complementary approach to stem cell therapies in clinical trials. Moreover, due to the extensive knowledge available on SCI rehabilitation, it is straightforward to design customized training tailored to patients’ particular impairments. This allows rehabilitation therapists to gain valuable input from patients’ prompt responses [52]. Rehabilitation training comes with drawbacks, including being less practical and costly and making long, drawn-out preclinical studies challenging. There is also the challenge that not many laboratories that are investigating regenerative treatment have the tools, infrastructure, and assets essential for executing rehabilitative techniques. Additionally, effective training procedures have yet to be authenticated or made uniform throughout research teams. Regularized guidelines for forelimb limb rehabilitation and four-legged treadmill practice have only recently been launched in rodent models with injury in the spinal cord. For that reason, assessing the findings of regenerative rehabilitation preclinical studies is complicated [14,22,52]. Deeper studies will support ways to make treadmill training and stem cell therapy combination more impactful while mitigating risks despite the limited documentation on the mechanism that is the reason for changes. Mechanisms are outlined in Figure 1 [52].

Over the last decade, the clinical application of regenerative medicine has emerged as a valuable tool to treat a variety of disorders. Stem cell transplantation offers hopeful prospects as a new therapeutic paradigm for stroke recovery. The long-term survival of transplanted cells depends on their ability to integrate into existing neural systems and generate the appropriate microenvironment. In order to achieve therapeutic success, it is critical to retrain the damaged native neural networks to recover physiological function. The incorporation of rehabilitation is a key component in improving the clinical translation of cell therapies and regenerative interventions for stroke, as outlined in Stem Cell Therapies as an Emerging Paradigm in Stroke (STEPS) [4,14,28]. However, certain points remain to be addressed before cell therapies can be converted into viable clinical products for stroke, particularly concerning the specific cellular outcomes of integrating rehabilitation in regenerative therapy; the potential synergistic or antagonistic effects of rehabilitation coupled with regenerative approaches; and the optimal dose, timing, and frequency of administration. Evolving clinical research in regenerative medicine is poised to bridge these knowledge gaps [3,22,28]. Intracerebral cell therapy has been recognized as a promising new therapeutic intervention for stoke treatment. The influence of physical therapy in relation to the implanted cells and their effectiveness in facilitating the healing process, however, is not fully known. The recommended course of treatment for stroke patients is multimodal physical therapy, which includes task integration, aerobic exercise, and resistance training. Enriched environments are used to emulate these multimodal dynamics in animal models for stroke research. In some studies, environmental enrichment was proven to be therapeutically beneficial when used in tandem with intravascular or intracerebral cellular therapy treatment, but in other studies, the outcomes were contradictory [4,14,54]. However, housing and enrichment conditions usually vary among laboratories; thus, it remains poorly understood which particular aspects of the experimental design impact therapeutic outcomes, creating inherent uncertainty in translational validity. On the other hand, aerobic exercise can be implemented using treadmills in both animal models and patients undergoing treatment for stroke. A significant majority (88%) of physical therapy professionals recommend aerobic exercise as a rehabilitative intervention for stroke patients. While aerobic exercise demonstrated additional benefits when combined with intravascular cell therapy for stroke, it did not enhance the effectiveness of NSCs following the insertion of an intracortical implant in a traumatic brain injury rat model. The potential interplay between these two complementary treatments and their influence on each other are important considerations for the practical application of NSC implantation for stroke treatment [14,54].

**Figure 2:**
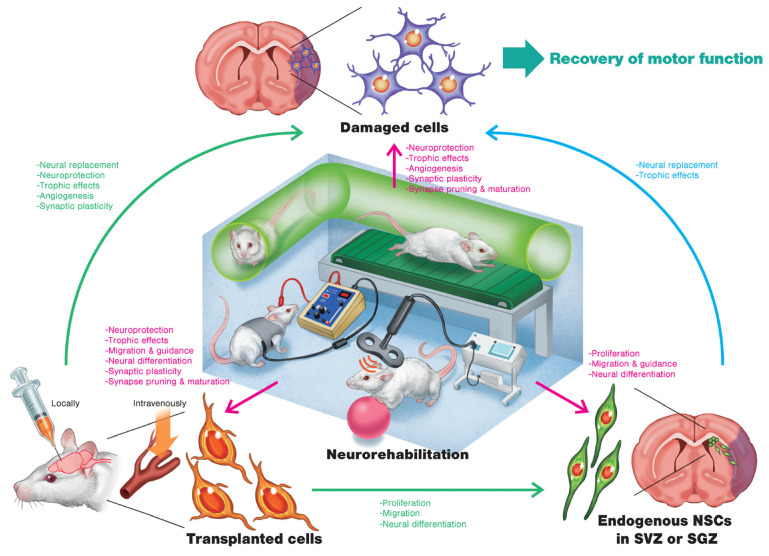
Schematic illustration of regenerative rehabilitation for stroke recovery in an animal model. Possible mechanisms of action related to cell therapy and neurorehabilitation are described. Transplanted cells affect endogenous neural stem cells (NSCs) in the subventricular zone or the subgranular zone, as well as damaged host cells in the infarct area. Neurorehabilitation, such as treadmill exercise, enriched environment interventions, repetitive transcranial magnetic stimulation (rTMS), and transcranial direct current stimulation, not only affects damaged cells but also affects endogenous NSCs and transplanted cells. Combining cell therapy and neurorehabilitation will be able to induce synergistic effects on motor function [28].

Cell transplantation is considered an attractive treatment option for stroke patients. The most frequently used cell delivery routes include intravenous and intraparenchymal transplantation, whereas MSCs, pluripotent stem cells, NSCs, NPCs, and umbilical cord blood cells are the cells used for transplantation. NSCs have the remarkable capacity to integrate into damaged host neural networks to replace lost cells, thereby promoting constitutive secretion of neurotrophic and anti-inflammatory factors, which enhance neurogenesis and tissue repair (Figure 2) [53]. Despite numerous clinical trials and ongoing research, regenerative therapy as an intervention for stroke recovery has not yet presented definitive outcomes or methodologies. Significant gaps in knowledge remain to be bridged before regenerative medicine can be established as an intervention for stroke treatment, particularly regarding the optimized delivery approach, timing, dosage, and selection of cell type and patient group. Furthermore, major challenges such as poor cell survival, engraftment issues, and control of differentiation need to be overcome [4,22,53]. Due to its multidimensional effectiveness, physical therapy has been advocated as a rehabilitative intervention both prior to and after hematopoietic stem cell transplantation (HSCT). Growing evidence demonstrates significant beneficial effects of incorporating physical therapy in strength, endurance, pulmonary capacity, and quality of life. However, in order to maximize the desired beneficial outcomes of HSCT, the type, frequency, and intensity of physical therapy interventions should be tailored and executed according to the individualized needs of patients [2,34,55].

Patients with multiple sclerosis experience debilitating disabilities, which significantly lower their quality of life. Although multiple sclerosis remains incurable, several therapeutic interventions, such as medication, stem cell transplantation, and physical therapy, can be utilized to mitigate symptom severity and improve the quality of life in people with multiple sclerosis. There is, however, limited research on the outcomes of combining therapeutic strategies for multiple sclerosis. Alghwiri et al. examined the consequences of pairing physical therapy exercises (PTEs) with Wharton’s jelly mesenchymal stem cells (WJ-MSCs) transplantation on mobility-related and other nonmotor symptoms, as compared to each respective intervention alone, in patients with multiple sclerosis [25,36,56]. A total of sixty patients with multiple sclerosis were allocated to groups receiving PTEs, WJ-MSCs cell therapy, or a combination of the two therapies, with a follow-up period of 12 months to monitor the effects comprehensively. Patients in the PTE group received biweekly sessions of a supervised exercise program for six months, followed by a home exercise program for the remaining six months of the study duration. The WJ-MSCs group were administered three WJ-MSCs injections during the first six months and then encouraged to adopt a healthy lifestyle for the remaining six months. The combined treatment group received a combination of both interventions. This strategy can potentially reduce disability and improve the quality of life for multiple sclerosis patients, thus contributing towards reducing the worldwide costs associated with providing these patients with lifetime care [56]. The safety and feasibility of physical therapy as a rehabilitative intervention for patients suffering from cytopenia during allogeneic hematopoietic stem cell transplantation (allo-HSCT) were investigated by Morishita and colleagues. They also explored how physical therapy impacts physiological outcomes and quality of life in these patients. A total of 321 patients who received allo-HSCT were recruited for the study. Participants were split into two groups, the physical therapy group (227 patients) and the control group (94 patients), in order to investigate the safety and feasibility of physical therapy in patients suffering from cytopenia. Patients in the physical therapy cohort were further allocated into subgroups according to the frequency of physical therapy received (51 patients in each group) in order to study the effects of physical therapy. Physiological function was assessed using a 6 min walk test (6MWT), handgrip strength, and knee extensor strength. The Short-Form 36 questionnaire was used to evaluate quality of life. Patients receiving physical therapy showed a higher rate of engraftment and lower incidence of mortality compared to the control group (*p* < 0.05). Following HSCT, significantly lower deterioration in physical functioning and quality of life was observed in the patients receiving high-frequency physical therapy as compared to patients in the low-frequency subgroup (*p* < 0.01). These results suggest that physical therapy has beneficial effects and can be implemented safely and feasibly in patients with cytopenia during HSCT [57]. A multitude of noninfectious respiratory complications may develop in patients within the first few weeks post-HSCT. Waked et al. determined the efficacy of chest physical therapy (CPT) given prior to transplantation in patients awaiting allo-HSCT by measuring spirometric values and respiratory muscle strength. A total of 50 patients, aged between 40 and 55 years and scheduled for allo-HSCT, were randomly divided into two groups: one group received CPT along with standard medical care, while the other group served as the control, receiving standard medical care only. Standard physical therapy was provided to both groups throughout the inpatient waiting period. Before undergoing allo-HSCT, the interventions were carried out every day for three weeks. Respiratory muscle strength was determined by a respiratory pressure meter, and pulmonary function (FEV1, FVC, and FEV1/FVC) was assessed by spirometry. For all variables being monitored, assessments were performed three weeks prior to allo-HSCT (T0), at the end of treatment right before starting allo-HSCT (T1), and three weeks following allo-HSCT (T2). The CPT group significantly outperformed the control group in terms of mean spirometric values and respiratory muscle strength, including maximal inspiratory pressure and maximal expiratory pressure, at both T1 and T2, with a significance level of *p* < 0.05. Incorporation of the three-week CPT intervention into the pretransplant regimen for HSCT recipients was safe and effective, as it enhanced pretransplant respiratory function and respiratory muscle strength while also preventing the deterioration of these functions after transplantation [58].

Ambrosio et al. investigated the effects of exercise on stem cell transplantation therapy in mice with skeletal muscle injuries. Results showed that five weeks of daily treadmill running significantly increased the number of transplanted cells. Additionally, it was found that the majority of the transplanted cells had undergone terminal differentiation towards myogenic lineages, whereas in the absence of mechanical stimulation from treadmill running, the transplanted cells were not able to divide quickly [59]. Yamaguchi and colleagues examined the histologic effect of treadmill running following intracranial transplantation of stem cells for the treatment of osteochondral defects in rats. Utilizing the Wakitani cartilage repair scoring system, results revealed that exercise significantly enhanced cartilage repair after stem cell transplantation, particularly at the 4-week mark. This research underscores the role of exercise post-transplantation in treating articular cartilage defects [60]. In order to treat idiopathic osteonecrosis of the femoral head, Aoyama and colleagues demonstrated the feasibility and safety of a 12-week rehabilitation strategy after MSCs transplantation combined with vascularized bone grafting. The study objectives were to promote bone growth from the implanted stem cells, prevent femoral head collapse, and improve hip joint function.

A combination of resistance training, aerobic exercises, progressive weight-bearing activities, and passive and active range-of-motion exercises was incorporated into the program, and the progression of these exercises during the program was based on the current scientific literature [61].

Given the intense nature of HSCT therapy, there is a risk of developing long-term side effects that may profoundly impair physical function and body composition post-transplantation. Takekiyo and colleagues examined the effects of exercise in allo-HSCT recipients by measuring muscle mass and physical functioning before and after the procedure. Participants included 86 patients who received allo-HSCT at Imamura Bun-in Hospital between February 2010 and September 2013. Exercise therapy was provided under the supervision of professional physical therapists five days a week, beginning two weeks prior to HSCT. Measurements for handgrip strength, body composition, and 6 MWT scores were recorded two weeks prior to and six weeks post-HSCT. Based on availability for both pre- and post-HSCT evaluations, thirty-five participants were included in the study. After receiving HSCT therapy, a significant decrease was observed in handgrip strength (*p* < 0.001) and 6MWT scores (*p* = 0.005). Despite a substantial reduction in upper extremity and trunk muscle mass (*p* = 0.001 and *p* < 0.001, respectively), the muscle mass of the lower extremities was notably unaffected after HSCT, suggesting that exercise may be effective in maintaining muscle mass of lower extremities in patients receiving HSCT [62]. Intracerebral cell therapy is gaining recognition as a novel treatment paradigm for stroke. The effects of physical therapy on implanted cells and their potential to facilitate the recovery process, however, are not well elucidated. Ghuman and colleagues conducted a study where a human NSC line was implanted into peri-infarct tissue using injection sites defined by MRI two weeks following a stroke. Physical therapy, in the form of aerobic exercise, was administered five times per week postimplantation, employing a regimen commonly utilized for stroke patients. When combined with cell therapy, aerobic exercise had a subadditive impact on sensory neglect. In fact, aerobic exercise with cell therapy had an antagonistic effect on motor integration and grip strength. Behavioral testing was essential to successful task integration. The study offers new insight into how to incorporate physical therapy into the design of clinical trials evaluating NSC implantation for the treatment of stroke [54].

### 4.3. Growth Factors Therapy

The histological outcomes mentioned above are driven by cellular and molecular processes encompassing trophic support and anti-inflammation. The expression of several neurotrophic factors is elevated as a secondary response to pharmacological and rehabilitative interventions. Brain-derived neurotrophic factor (BDNF), insulin-like growth factor-1, glial cell line-derived neurotrophic factor, neurotrophin 3, and neurotrophin 4, which play major roles in the survival, maintenance, and maturation of neurons, are upregulated as a result of treadmill exercise. Similarly, it is reported that repetitive transcranial magnetic stimulation (rTMS) induces the production of BDNF and the expression of nerve growth factor. In contrast, intermittent rTMS upregulates epidermal growth factor and basic fibroblast growth factor [5,6,27,52]. Cyclical loading promotes physiological cartilage homeostasis, which is party regulated by mechanical activation of transforming growth factor beta (TGF-β) present in the extracellular matrix. In native cartilage, this may strongly be influenced on a mechanical level in the superficial zone but regulated enzymatically within deeper zones [1,7,12]. Human bone marrow-derived MSCs, such as those induced by microfracture, can be stimulated by mechanical force to differentiate into chondrocytes under specific in vitro conditions. Similar responses have also been demonstrated for human articular chondroprogenitor cells. Differentiation stimulated by mechanical force occurs due to the induction of endogenous TGF-β production by the application of shear force. Rehabilitation protocols should be designed based on the underpinning scientific rationale, taking into account the intricacies of mechanical interplay at the cellular and molecular levels [1]. TGF-β is conventionally administered in regenerative medicine protocols for cartilage regeneration. The dosage and timing of TGF-β administration may critically impact regeneration; however, there is limited research into the optimization of TGF-β treatment. The conventional in vitro induction medium for chondrogenic cell culture is typically supplemented with 10 mg/mL TGF-β. Over the years, numerous studies have evaluated the use of bioreactors in the mechanical regulation of chondrogenesis of human MSCs. A bioreactor can apply compression force, shear stress, or a combination of both by rotation of a ceramic hip ball along the surface of a construct. Combining compression and shear forces when using bovine articular chondrocytes can result in outcomes that are histologically more similar to native cartilage. Similarly, it has been reported that even in the absence of exogenous serum or growth factors, the application of a combination of compression and shear forces results in the mechanical induction of chondrogenic differentiation of human MCSc [16,17,63]. The use of compression force alone under the same conditions yielded no response. Variations in frequency and amplitude of application of compression and shear forces affected chondrogenic differentiation, which is partly due to the activation of endogenous TGF-β by mechanical stimulation. TGF-β activation is also possible in cell-free scaffolds, underscoring that mechanical force, alone or in part, is responsible for the induction of TGF-β activation. It has been shown that mechanical shearing of synovial fluid can trigger the activation of TGF-β in large amounts, possibly due to the cleavage of the latency-associated peptide from the TGF-β by mechanical force. In response to mechanical load, it is proposed that the localized activation of TGF-β may be a mechanism for the onset of localized strain, resulting in localized biological outcomes [16,17,23,24,63]. TGF-β activation seems to be dependent on contractile forces and stiffness of the material under loading. Lower stiffness of materials significantly attenuates the induction of TGF-β activation because biomaterials that are “too soft” are unable to support the activation of cellular proteins. As loads are applied onto scaffolds and defects, building on this knowledge is necessary for the design of novel biomaterials that can activate latent reserves of TGF-β under mechanical load. There is also a further need for clinical trials to investigate the role of application of localized strain on TGF-β activation [12,25,63]. Platelet-rich plasma (PRP) is a commonly used application of regenerative therapy in orthopedic sports medicine, particularly for the treatment of ligament and tendon injuries. Although the concept of PRP therapy was first introduced in the 1970s, interest in its use in regenerative medicine for the treatment of musculoskeletal injury and disease has significantly risen over the years. Rehabilitation interventions, such as physical therapy, are often used in conjunction with PRP. However, due to a lack of clinical data and little information available regarding optimal protocol design, scientific consensus has not been reached on the incorporation of rehabilitation with PRP therapy [16].

PRP acts as a natural reservoir of growth factors, which exert anti-inflammatory effects and stimulate intrinsic regeneration pathways, underscoring its great potential in regenerative medicine. However, in the absence of an appropriate drug delivery system, direct injection of PRP often results in the wastage of therapeutic material due to leakage, diffusion, denaturation, or circulatory clearance. The purpose of delivery systems is to carry therapeutic agents to the target sites, followed by their release in a more controlled manner [6,12,13]. Material composition and structural features of delivery systems can be modified to exert control over desired release patterns. Controllable release of growth factors, cytokines, nucleotides, MSCs, and exosomes have unparalleled potential in regenerative medicine due to their proregenerative properties and ability to promote tissue regeneration. Matrilin-3, a noncollagenous ECM protein, is involved in cartilage development and ossification. A study showed that the application of Matrilin-3 to extracellular matrix scaffolds can improve articular cartilage regeneration with BMSCs by maintaining chondrogenesis and inhibiting chondrocyte hypertrophy [13,17]. A case series presented a five-phase rehabilitation protocol for patients of patellar tendinopathy treated with PRP therapy. The target of the first three stages was to create optimal conditions for the promotion of recovery. In comparison, the last two stages were designated for physical activity, with the progressive increase in intensity of interventions, in reference to accurate performance of the exercises and a visual score not exceeding 50, quantified on a 0–100 range analog scale. In the third phase, eccentric exercises were incorporated into the rehabilitation program, initially performed biweekly, allowing the tendon to acclimatize to the magnitude of the load [64].

In order to establish a relationship between mechanical stimulation and post-PRP recovery, Virchenko and Aspenberg investigated the effect of PRP therapy in healing Achilles tendons of rats, with and without mechanical load. Results showed positive effects on biomechanical characteristics, while PRP also enhanced the material properties of tissue healing. However, the desired outcomes were short-lived when the mechanical load was removed. The authors concluded that rehabilitation may potentially be critical in ensuring the success of PRP therapy. Results also showed the influence of platelets in only the early stages of tendon regeneration, underscoring the role of mechanical stimulation in the early phases of the regenerative healing process [65]. Kaux et al. proposed a standardized 6-week rehabilitation strategy based on submaximal eccentric therapy for patients receiving PRP therapy for patellar tendinopathy. A week of relative rest is prescribed before beginning a progressive routine, which is performed thrice a week, consisting of closed kinetic chain exercises for strengthening the quadriceps muscles [66]. A recent study demonstrated the effects of intensive quadrupedal treadmill exercise in reducing inflammation in rats receiving GABAergic NPCs transplantation therapy for spinal cord injury. An increase was observed in the anti-inflammatory marker IL-4 in cerebrospinal fluid, while the proinflammatory mediators TNF-α and interleukin 1β were significantly reduced following training [67]. Death of transplanted NSCs can be triggered by the induction of reactive oxygen or reactive nitrogen species, although the mechanism behind this remains largely unknown. A study shows that treadmill rehabilitation attenuates cellular stress generated by reactive oxygen and reactive nitrogen species through the upregulation of IGF-1 signaling. It presents the only documented evidence of a causal relationship between certain cellular events and their subsequent effects on rehabilitative interventions combined with regenerative therapy [68]. 

## 5. The Combination of Rehabilitation and Regeneration

Only a few published case reports and series have studied the effects of regenerative therapies for the treatment of volumetric muscle loss (VML) in injuries. For these studies, participants received rehabilitative physical therapy prior to and postsurgical transplantation of an extracellular matrix (ECM). In all cases, rehabilitative intervention on its own did not improve desired outcomes [12,16,17,69,70,71]. The first clinical application of a decellularized ECM scaffold in tissue engineering was performed on a wounded soldier presented with a large VML of the quadriceps muscles of the thigh as a result of an improvised explosive devise blast. The patient had received latissimus dorsi muscle flap treatment for defects associated with loss of soft tissue due to an open fracture, successfully leading to the limb being salvaged. However, almost three years postinjury, the patient showed a 72% loss of isokinetic knee extensor strength despite receiving extensive conventional physical therapy for over a year and a half postinjury. Four months following ECM implant therapy coupled with physical therapy designed to recover knee function, an improvement of 13% of knee extensor strength was observed using an isokinetic dynamometer [5,6,12,27,69,70]. However, a 68% strength deficit had been retained following the experimental procedure. In a case series, five patients received ECM implants in the region of VML and were prescribed a rehabilitative physical therapy regimen designed specifically for recovery from injury. The VML injuries had resulted in approximately 60–90% loss of contralateral limb musculature and were localized in the quadriceps or anterior tibial compartments in the lower limbs of the patients. Three of the five patients showed approximately 20–136% improvement in isometric muscle strength of the injured limb, measured using a handheld dynamometer, while no changes were observed in the two other participants. As preinjury measurements had not been recorded, it is impossible to assess the strength deficit in this study. As a follow-up to Sicari et al., a case study described a rehabilitative physical therapy program for a patient with a surgical ECM implant for the treatment of quadriceps VML. After 27 weeks following ECM transplantation and rehabilitation, a 20% increase in isometric knee extensor strength was observed. Prior to surgery, there was an 89% strength deficit in the operated leg compared to the unoperated limb, which decreased to 87% at the 27-week mark post-transplantation [12,16,24,69,70].

## 6. Academic Rehabilitation Programs Incorporating Regenerative Medicine

At its core, the primary aim of regenerative rehabilitation is to regenerate damaged tissue or organs and restore physiological function by integrating regenerative cell therapies with physical rehabilitation practices. Regenerative rehabilitation spans the use of cell therapies, pharmacological interventions, and bioengineering products coupled with physical rehabilitation to restore function and improve the quality of life in people living with disabilities [27,72]. The Department of Rehabilitation and Regenerative Medicine, set up by Dr. Joel Stein in 1952 at Columbia University in New York, is the pioneering academic department combining regenerative medicine with rehabilitation. The Department of Rehabilitation and Regenerative Medicine hosts the Columbia Stem Cell Initiative, which oversees more than 100 stem cell laboratories [72]. Dr. Christopher Henderson of the Department of Rehabilitation and Regenerative Medicine is the director of the Stem Cell Initiative, which serves to advance stem cell research and provide continuous education and training to the stem cell community. Combining stem cell research with rehabilitation under the same academic department offers an attractive opportunity to broaden our understanding of the underlying biological principles of rehabilitative interventions. The Rehabilitation Medicine Research Center, directed by Dr. Christopher Evans, was launched by the Department of Physical Medicine and Rehabilitation at the Mayo Clinic [72]. The central goal of this center, overseen by Dr. Jay Smith of the Department of Physical Medicine and Rehabilitation and the Centre for Regenerative Medicine at the Mayo Clinic, is to draw on clinical research to design multidisciplinary programs focused on innovation and the translation of applications in regenerative rehabilitation, accelerating the transfer of cutting-edge research regarding diseases and their cures to improve the quality of care in clinical practices. Across the U.S., many other university academic departments are following suit through collaborations with rehabilitation medicine centers for the convergence of rehabilitation with regenerative technologies. At the University of Pittsburgh, the McGowan Institute for Regenerative Medicine is in a collaborative partnership with the Department of Rehabilitation Medicine for research, which ranges from musculoskeletal regeneration to advancements in brain–computer interface technology for repair and rehabilitation [6,16,27,72]. The Institute for Stem Cell and Regenerative Medicine at the University of Washington is also in collaboration with the Department of Rehabilitation Medicine, whose researchers are facilitated through prioritization in the allocation of primary working space in cutting-edge stem cell research laboratories. Rehabilitation scientists at this facility are involved in projects encompassing both genetic therapy and stem cell research. Regenerative therapy is an emerging field that is poised to usher in a new era in medicine, with the primary focus on the restoration and repair of physiological function in organs damaged due to trauma, disorder, and disease. Unraveling the underlying biological principles of incorporating rehabilitation into regenerative medicine is imperative to accelerate the translation of evidence-based research into clinical practices, which have the potential to convert the idea of “care” into “cure”. Readers will come across multiple examples in this supplement that illustrate the use of regenerative rehabilitation in clinical practice, thereby bridging the theory–practice gap [16,27,72].

## 7. Limitations

Regenerative medicine holds promising potential, although several hurdles remain to be overcome before clinical application becomes a possibility. Invasiveness and special requirements: Techniques in regenerative medicine are often invasive, requiring specialized equipment and optimized conditions. This significantly limits their widespread clinical implementation. Variable efficacy: The efficacy of regenerative treatments varies significantly across studies. Variations depend on numerous factors including study design, patient population, genetic makeup, age, sex, weight, health status, and condition severity. These discrepancies make it challenging to compare results across trials and establish best practices for clinical implementation. Regulatory challenges: Clinicians and researchers face difficulties navigating the complex and rapidly evolving regulatory framework for developing rehabilitation therapies. Seeking regulatory approval for regenerative therapies can be a lengthy and costly process, delaying patient access to these treatments. Ethical concerns: There are moral considerations regarding the fair allocation of regenerative therapies and the risk of exploiting vulnerable populations for components. These ethical issues need to be addressed to ensure equitable treatment distribution. Integration with physical therapy: The optimal integration of regenerative techniques with conventional physical therapy is still under exploration. Consequently, there is limited information on the clinical applications of these combined therapies within existing therapeutic paradigms. Collaborative efforts: Collaborative efforts between rehabilitation scientists and regenerative medicine specialists are essential for developing treatment strategies tailored to the multifaceted needs of each patient. However, there is currently a lack of knowledge about the long-term sustainability of regenerative therapies. Scalability and cost: Questions remain regarding the scalability of regenerative treatments and their potential impact on healthcare costs and resource allocation. These concerns must be addressed to ensure the practical and economic feasibility of widespread regenerative medicine use. Workforce expertise: The advancement of regenerative medicine relies heavily on a well-trained workforce. Effective utilization of the latest regenerative techniques, in conjunction with traditional physical therapy interventions, requires specialized training and education. These limitations highlight the need for continued research and development to overcome the barriers to the clinical application of regenerative medicine, ensuring its effective integration with physical therapy for comprehensive patient care.

## 8. The Prospects and Future Directions

Regenerative medicine is an interdisciplinary field that combines tissue engineering with stem cell therapy, materials science, and developmental biology and has the potential to revolutionize the future of medicine. As research continues to unravel the intricacies of cellular and molecular mechanisms driving the therapeutic effects of regenerative therapies, the integration of physical therapy into regenerative medicine offers significant opportunities to improve desired outcomes and quality of life. Enhanced tissue repair and regeneration: In the field of regenerative medicine, techniques such as stem cell transplantation and growth factor administration have demonstrated significant potential in tissue regeneration and repair. Future research efforts must focus on the standardization of regenerative therapies to optimize their efficacy and safety, thus accelerating their translation to the clinic for the treatment of neurological disorders, degenerative joint diseases, and musculoskeletal injuries or disorders. Continuous advancements in scaffold design, biocompatible materials, and controlled delivery systems are expected to play a pivotal role in facilitating the targeted delivery of therapeutic agents to affected tissues, promoting recovery, and mitigating symptoms of debilitating conditions. Personalized treatment modalities: With the advent of precision medicine, the future of regenerative medicine in physical therapy is poised to embrace personalized treatment modalities based on the individual medical needs or subgroup characteristics of each patient. The integration of biotechnological platforms, including genetic testing, biomarker analysis, and imaging techniques, will enable early detection and monitoring of diseases, allowing clinicians to customize therapeutic strategies tailored to the unique biological makeup and pathological features of each patient. With personalized approaches underscoring optimized treatment outcomes, minimal adverse effects, and heterogeneity of diseases, a path is paved toward more effective and patient-centric care paradigms. Personalized medicine holds the potential to usher in a new era in medicine by accelerating the healing process and managing outcomes in patients with various musculoskeletal disorders, including injury. Innovations in rehabilitation strategies: Along with facilitating tissue repair, regenerative medicine also holds the potential to revolutionize rehabilitative medical interventions through the incorporation of advanced technologies and modalities. Pioneering approaches in tissue engineering and bioelectronic medicine, such as 3D bioprinting, offer the opportunity to create functional tissue constructs, augment neuromuscular control, and restore mobility in patients with disabilities. Furthermore, the intersection of regenerative therapies and robotic-assisted rehabilitation, including the use of wearable devices, artificial intelligence, and virtual reality training, is anticipated to redefine the rehabilitation landscape by enabling personalized, interactive, and immersive interventions individualized specifically to the capability and needs of each patient. Addressing ethical and regulatory challenges: As regenerative medicine continues to evolve at a rapid pace, it is imperative to address the ethical, legal, and regulatory challenges associated with its implementation in clinical practice. Ethical considerations associated with the sourcing and utilization of stem cells, the safety of experimental therapies, and equitable access to innovative treatments necessitate careful scientific deliberation and regulatory oversight. Moreover, fostering public trust by ensuring access to information, transparency, informed consent, and patient autonomy is essential to upholding ethical standards in regenerative care. Collaborative efforts among policymakers, healthcare professionals, bioengineers, and bioethicists are essential to establish robust regulatory frameworks that balance innovation with patient safety and ethical considerations. Translational research and clinical translation: The translation “bench to bedside” of regenerative therapies hinges upon robust clinical research aimed at bridging the gap between preclinical studies and clinical applications. Long-term clinical trials, multicenter collaborations, and real-world evidence studies are indispensable for evaluating the long-term safety and efficacy of outcomes in diverse patient populations. Furthermore, fostering a collaborative interdisciplinary ecosystem that nurtures innovation and knowledge exchange can accelerate the adoption of regenerative medicine therapies in mainstream clinical practice, thereby transforming the landscape of healthcare and physical therapy. Patient education and empowerment: In the era of personalized medicine and shared clinical decision making, patient education and empowerment comprise a cornerstone of effective regenerative interventions in physical therapy. Educating patients about the underlying mechanisms and potential benefits and risks associated with regenerative therapies is essential for informed decision making and active engagement in their treatment journey. Moreover, providing patients with the necessary tools, resources, and support networks enhances adherence to protocols, facilitates self-management strategies, and fosters a sense of ownership and autonomy over their health. By prioritizing patient-centered care and fostering collaborative partnerships between patients and clinicians, the desired outcomes can be optimized and can improve the overall patient experience in the realm of regenerative medicine and physical therapy.

## 9. Conclusions

The integration of regenerative medicine into physical therapy practices represents a paradigm shift in healthcare, particularly in the domains of neurological and musculoskeletal rehabilitation. This convergence offers unprecedented opportunities to enhance tissue repair, personalize treatment modalities, and innovate rehabilitation strategies. By combining the regenerative potential of therapies such as stem cell treatments, platelet-rich plasma, and gene therapy with traditional physical therapy techniques, patient outcomes can be significantly improved. Furthermore, fostering interdisciplinary collaborations between physical therapists, regenerative medicine specialists, and other healthcare providers is crucial. This collaborative approach ensures comprehensive patient care and maximizes the therapeutic potential of combined treatments. Prioritizing patient-centric care while adhering to ethical and regulatory standards is essential in navigating this evolving landscape. Ultimately, the integration of regenerative medicine into physical therapy not only enhances the healing and restoration process but also empowers patients in their journey toward recovery and resilience. By embracing these advancements, the field can move toward a new era of effective, innovative, and holistic rehabilitation practices.

## Data Availability

This is a review article, and all data are included in this text.

## Abbreviations

Extracellular matrix (ECM), central nervous system (CNS), brain-derived neurotrophic factor (BDNF), low-intensity vibration (LIV), polycaprolactone (PCL), bone marrow-derived mesenchymal stem cells (BMSCs), mesoporous bioactive glass (MBG), vascular endothelial growth factor (VEGF), spinal cord injury (SCI), neural stem cells (NSCs), neural progenitor cells (NPCs), mesenchymal stem cells (MSCs), olfactory ensheathing cells (OECs), Stem Cell Therapies as an Emerging Paradigm in Stroke (STEPS), post-hematopoietic stem cell transplantation (HSCT), pairing physical therapy exercises (PTE), Wharton’s jelly mesenchymal stem cells (WJ-MSCs), brain-derived neurotrophic factor (BDNF), transforming growth factor beta (TGF-β), volumetric muscle loss (VML).
